# Morbillivirus Infections: An Introduction

**DOI:** 10.3390/v7020699

**Published:** 2015-02-12

**Authors:** Rory D. de Vries, W. Paul Duprex, Rik L. de Swart

**Affiliations:** 1 Department of Viroscience, Erasmus MC, Rotterdam 3000, The Netherlands; E-Mail: r.d.devries@erasmusmc.nl; 2 Department of Microbiology, Boston University School of Medicine, Boston 02118, MA, USA; E-Mail: pduprex@boston.edu

**Keywords:** paramyxovirus, morbillivirus, measles, rinderpest, distemper, vaccination

## Abstract

Research on morbillivirus infections has led to exciting developments in recent years. Global measles vaccination coverage has increased, resulting in a significant reduction in measles mortality. In 2011 rinderpest virus was declared globally eradicated – only the second virus to be eradicated by targeted vaccination. Identification of new cellular receptors and implementation of recombinant viruses expressing fluorescent proteins in a range of model systems have provided fundamental new insights into the pathogenesis of morbilliviruses, and their interactions with the host immune system. Nevertheless, both new and well-studied morbilliviruses are associated with significant disease in wildlife and domestic animals. This illustrates the need for robust surveillance and a strategic focus on barriers that restrict cross-species transmission. Recent and ongoing measles outbreaks also demonstrate that maintenance of high vaccination coverage for these highly infectious agents is critical. This introduction briefly summarizes the most important current research topics in this field.

## 1. Introduction

The genus *Morbillivirus* belongs to the virus family *Paramyxoviridae*, a group of enveloped viruses with non-segmented, negative strand RNA genomes. It contains viruses that are highly infectious, spread via the respiratory route, cause profound immune suppression, and have a propensity to cause large outbreaks associated with high morbidity and mortality in previously unexposed populations. In populations with endemic virus circulation, the epidemiology changes to that of a childhood disease, as hosts that survive the infection normally develop lifelong immunity. 

**Figure 1:**
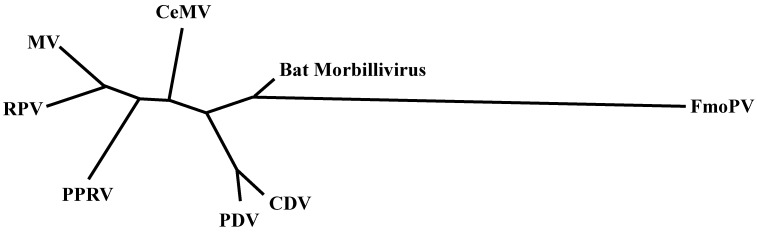
An unrooted maximum likelihood phylogenetic tree of morbilliviruses was estimated under the general time-reversible model using PhyML software version 3.0 [5], using partial large (L) gene nucleotide sequences (GTR + I + G model).

Measles virus (MV) is the prototype morbillivirus, and causes disease in primates. Rinderpest virus (RPV) is closely related to MV and used to cause severe disease in cattle. Other viruses in the genus *Morbillivirus* include peste des petits ruminants virus (PPRV), which causes disease in small ruminants, such as goats and sheep; canine distemper virus (CDV), which causes distemper in dogs and a large number of other carnivore species; phocine distemper virus (PDV), which leads to distemper in several seal species and cetacean morbilliviruses (CeMV), which cause disease in dolphins and whales. As shown in Figure 1, all morbilliviruses are phylogenetically closely related [1,2]. Recently, a newly described virus of cats was named feline morbillivirus (FmoPV) [3]. Whether FmoPV is a “true” morbillivirus remains to be determined: Both molecular biology and inferred pathogenesis suggest that this is not the case. Finally, sequences of a morbillivirus endogenous to neotropical vampire bats were found in Brazil, potentially representing a new member of the genus *Morbillivirus* [4]. Yet again, the detection of short sequences does not equate to the presence of infectious virus, and efforts should be made to isolate pathogens where possible.

## 2. Measles

Measles remains a significant cause of childhood morbidity and mortality in humans. The disease is characterized by fever, skin rash, cough and conjunctivitis, and a generalized immune suppression [6]. The resulting increased susceptibility to opportunistic infections may lead to life-threatening complications such as pneumonia and/or gastro-intestinal disease. Safe and effective live-attenuated virus vaccines have led to a substantial reduction in measles morbidity and mortality [7]. However, numbers of measles deaths have increased again in 2014, and measles resurgence may be imminent due to reduced measles vaccine acceptance in the industrialized world and budget deficits to maintain vaccination coverage in developing countries [8].

## 3. Rinderpest

Within the *Morbillivirus* genus, MV is most closely related to RPV. It has been postulated that RPV or a closely related ancestral cattle virus crossed the species barrier into humans 1000–5000 years ago [2]. At the end of the 19th century, introduction of RPV into previously unexposed cattle resulted in catastrophic outbreaks, causing decimation of herds and famine in many African countries [9,10]. In consecutive decades, RPV had a devastating impact on cattle worldwide and the human populations that depended on them. Through the use of an efficacious vaccine rinderpest virus was declared globally eradicated in 2011 [11].

PPRV is referred to as “the plague of small ruminants” and is a highly contagious and fatal disease for sheep and goats. Being closely related to RPV, and considering the importance of sheep and goats to farmers in Africa and South Asia, PPRV may also become a potential candidate for global eradication [12]. A comprehensive review of PPRV can be found in this special issue of *Viruses* [13].

## 4. Distemper

Canine distemper has been described as an infectious disease of dogs since the 17th century, but CDV has the capacity to infect a wide range of carnivores. Since distemper is associated with high morbidity and mortality [14], most domestic carnivores are vaccinated. CDV infection can obliterate virtually all lymphocytes in a host, leading to severe immune suppression. Interestingly, from an epidemiologic point of view CDV is unlike any other morbillivirus, since it can be sustained in target species living in low-density populations. In addition, the virus is renowned for its neurovirulence. Both characteristic aspects of this disease may be related to the severity of the immune suppression and the resulting prolonged virus shedding. A review of the pathogenesis of CDV leukoencephalitis can be found in this issue [15].

## 5. Marine Mammal Morbilliviruses

PDV and CeMV, two phylogenetically distinct morbilliviruses, have caused significant disease outbreaks in marine mammals. PDV mainly infects seals, and two large outbreaks have been reported thus far. In 1988, approximately 18,000 harbor seals died in northwest Europe. A second, and equally devastating outbreak, occurred in the same area in 2002 [16]. Notably, disease outbreaks in seals in Lake Baikal in 1987 and in the Caspian Sea in 2000 were not attributed to PDV, but to CDV [17,18]. It is estimated that currently only 11% of the seals in Dutch coastal waters have PDV-specific antibodies, meaning that a re-introduction of PDV or CDV into this population could spark another outbreak with equivalent mass-mortality [19].

Over the last three decades CeMV has been responsible for large disease outbreaks among cetaceans, including harbor porpoises, striped dolphins, bottlenose dolphins, and pilot whales. Two comprehensive reviews of morbillivirus infections in seals and cetaceans can be found in this issue [20,21].

## 6. Other Morbilliviruses

Recently, novel morbilliviruses were detected in felines and bats. FmoPV was first described in 2012 as a morbillivirus of cats, and has thus far only been identified in China and Japan. Phylogenetically FmoPV is distantly related to the other morbilliviruses [3]. In addition, the pathogenesis of FmoPV is different from the other morbilliviruses. FmoPV has been associated with tubulointerstitial nephritis, making it questionable whether FmoPV is a “true” morbillivirus. In Brazilian vampire bats two short morbillivirus polymerase sequences were discovered in 2012. However, no infectious virus has been isolated [4]. Whether these sequences represent novel morbilliviruses endemic among bat populations remains to be determined.

## 7. Cellular Receptors and Cross-Species Infections

Infection of cells by morbilliviruses is initiated by binding of the hemagglutinin (H) glycoprotein to cellular receptors. Two cellular receptors have been described for wild-type morbilliviruses: CD150 (also known as signaling lymphocyte activation molecule F1 or SLAM/F1) in 2000 [22] and poliovirus receptor-like 4 (PVRL4, also known as nectin-4) in 2011 [23,24]. CD150 is mainly expressed on subsets of immune cells, while PVRL4 is expressed in epithelial cells. Since morbilliviruses can also infect cells of the central nervous system, additional receptors may exist. A review of the discovery of PVRL4 as cellular receptor for morbilliviruses can be found in this issue [25].

Interestingly, the phylogenetic relationships of the morbilliviruses largely parallel those of their respective host species [2,26]. The different morbilliviruses most likely evolved from a common ancestral virus that has adapted to their respective mammalian hosts, indicating that morbilliviruses have an intrinsic capacity to adapt to new host species [26]. This adaptation requires mutations in the morbillivirus receptor-binding H glycoprotein. The fact that the protein uses overlapping regions to bind to CD150 and PVRL4 [27] is likely a major hurdle for morbilliviruses to cross the species barrier.

Of all the morbilliviruses CDV is the most promiscuous, infecting many different carnivore species. Even though initially less pathogenic in new carnivore hosts, the virus caused large disease outbreaks in phocids and felids [28]. CDV also has the capacity to cross the species barrier from carnivores into primates: Several CDV outbreaks in non-human primates have been reported [29,30] and therefore the virus also may poses a potential threat for morbillivirus-naive humans. However, morbilliviruses induce cross-protection [31], and measles vaccination provides protection against CDV infection [32].

## 8. Intervention Strategies

Vaccination is the most effective intervention strategy to combat morbillivirus infections. Morbilliviruses are even considered as vectors for protection against other infectious diseases [33]. In recent years antiviral compounds have also been developed as post-exposure prophylaxis for high-risk contacts of confirmed index cases [34]. Such compounds could also be considered for use in endangered or captive wildlife species. In particular, both pre- and post-exposure treatment of ferrets with an orally available small-molecule polymerase inhibitor showed efficacy against CDV infection in ferrets [35]. Similarly, fusion-inhibitory peptides were shown to inhibit or prevent MV infection in small animal models [36].

## 9. Oncolytic Virotherapy

In recent years, MV has extensively been tested as a potential oncolytic virus [37]. These therapies either use the inherent lymphotropic nature of the virus, or try to retarget the virus to other cell types. In addition, PVRL4 is a well-known tumor-associated marker for several adenocarcinomas [25]. Viruses can be reprogrammed to improve their oncolytic potential, referred to as targeting (introducing multiple layers of cancer specificity to improve safety and efficacy), arming (expression of prodrug convertases and cytokines) and shielding (protection from immune responses) [38]. Interestingly, morbillivirus provides substantial genetic flexibility due to their pleomorphic nature [39]. A review of the potential of CDV as an oncolytic virus can be found in this special issue [40].

## 10. Conclusions

The identification of cellular receptors and improvement of animal models has provided important new insights into the pathogenesis of morbillivirus infections. It has become clear that all morbilliviruses initially infect cells of the immune system, before they spread to epithelial, endothelial and/or neuronal cells. Morbilliviruses remain a potential cause of disease outbreaks in previously unexposed populations. However, they can also be used to our advantage, as vaccine vectors or as oncolytic viruses. Sustained vaccination coverage and surveillance of circulating morbilliviruses will remain of critical importance for years to come [41].
